# Phase Space Generation for Proton and Carbon Ion Beams for External Users’ Applications at the Heidelberg Ion Therapy Center

**DOI:** 10.3389/fonc.2015.00297

**Published:** 2016-01-11

**Authors:** Thomas Tessonnier, Tiago Marcelos, Andrea Mairani, Stephan Brons, Katia Parodi

**Affiliations:** ^1^Department of Radiation Oncology, Heidelberg University Clinic, Heidelberg, Germany; ^2^Department of Medical Physics, Ludwig Maximilians University, Munich, Germany; ^3^Heidelberg Ion Beam Therapy Center, Heidelberg, Germany; ^4^Centro Nazionale di Adroterapia Oncologica, Pavia, Italy

**Keywords:** phase space, particle therapy, Monte-Carlo, FLUKA, patient dose calculation, experimental measurements

## Abstract

In the field of radiation therapy, accurate and robust dose calculation is required. For this purpose, precise modeling of the irradiation system and reliable computational platforms are needed. At the Heidelberg Ion Therapy Center (HIT), the beamline has been already modeled in the FLUKA Monte Carlo (MC) code. However, this model was kept confidential for disclosure reasons and was not available for any external team. The main goal of this study was to create efficiently phase space (PS) files for proton and carbon ion beams, for all energies and foci available at HIT. PSs are representing the characteristics of each particle recorded (charge, mass, energy, coordinates, direction cosines, generation) at a certain position along the beam path. In order to achieve this goal, keeping a reasonable data size but maintaining the requested accuracy for the calculation, we developed a new approach of beam PS generation with the MC code FLUKA. The generated PSs were obtained using an infinitely narrow beam and recording the desired quantities after the last element of the beamline, with a discrimination of primaries or secondaries. In this way, a unique PS can be used for each energy to accommodate the different foci by combining the narrow-beam scenario with a random sampling of its theoretical Gaussian beam in vacuum. PS can also reproduce the different patterns from the delivery system, when properly combined with the beam scanning information. MC simulations using PS have been compared to simulations, including the full beamline geometry and have been found in very good agreement for several cases (depth dose distributions, lateral dose profiles), with relative dose differences below 0.5%. This approach has also been compared with measured data of ion beams with different energies and foci, resulting in a very satisfactory agreement. Hence, the proposed approach was able to fulfill the different requirements and has demonstrated its capability for application to clinical treatment fields. It also offers a powerful tool to perform investigations on the contribution of primary and secondary particles produced in the beamline. These PSs are already made available to external teams upon request, to support interpretation of their measurements.

## Introduction

In the particle therapy field, Monte-Carlo (MC) codes provide a powerful tool to perform accurate calculations, with a precise description of the transport and interactions of the beam with the traversed materials, compared to the current treatment planning systems (TPS) as the one used at Heidelberg Ion Therapy Center (HIT; Syngo RT Planning TPS, Siemens AG Healthcare), which is based on analytical algorithms using fast pencil-beam dose calculation (1). At HIT, the FLUKA MC code (2, 3) was chosen to support the creation of the TPS basic input data (4). The beamline has been modeled in great details, particularly the vacuum window and the Beam and Application Monitoring System (BAMS), composed of two multiwire proportional chambers and three ionization chambers that are monitoring the beam, providing accurate data for the parameterization of the lateral dose spread for additional input to the analytical clinical TPS (5). A MC framework, without using the beamline model but a beamline approximation closely resembling the TPS approach, has been also developed and is used to perform both dose forward calculation and range verification (6–8), providing a powerful computational tool to complement the clinical TPS.

The use of modeled beamlines in MC applications has been described in many works for beam delivery with active energy selection (5, 9–11), for passive energy selection with pencil-beam scanning (12, 13), or for passive scattering (14, 15). In our case, due to confidential issues with the beamline geometry, the model is not available for external users in need of precise simulation, neither for data analysis comparisons after irradiation at HIT nor for simulation-related researches.

This paper proposes a solution to this problem with the creation of phase space (PS) files containing the characteristics (charge, mass, energy, coordinates and direction cosines, generation) of every particles (primary protons and carbon ions as well as secondaries) at the end of the beamline, for each of the 255 available initial beam energies. Furthermore, the adaptation to the delivery pattern from the raster scanning system (16) has to be possible with these PSs, as well as the accommodation of the four different foci used clinically at HIT, i.e., the full-width half maximum (FWHM) of the lateral beam sizes in air at isocenter according to the accelerator database (the so-called library of ion beam characteristics or LIBC). PS files created from beamline geometries, in the particle therapy field, have already been investigated for proton beam applications with passive beam delivery or scanned beams of fixed lateral size (13, 15, 17–19). Our approach proposes a novel narrow-beam approximation to generate PS that can be accurately adapted to reproduce all the foci available at HIT and scanning pattern of irradiation plans for both protons and carbon ions. Several validations steps against simulation with the full beamline geometry will be presented. Simulations using the PS approach will be compared to measurements in a water phantom. An application of the proposed approach to a small target patient plan will be shown and compared to the results of the simplified MC framework. For this plan, the two approaches will be evaluated against measurements in a water phantom.

## Materials and Methods

### Phase Space Generation

#### Monte-Carlo Code and Modeling Approaches of the HIT Beamline

**Figure 1:**
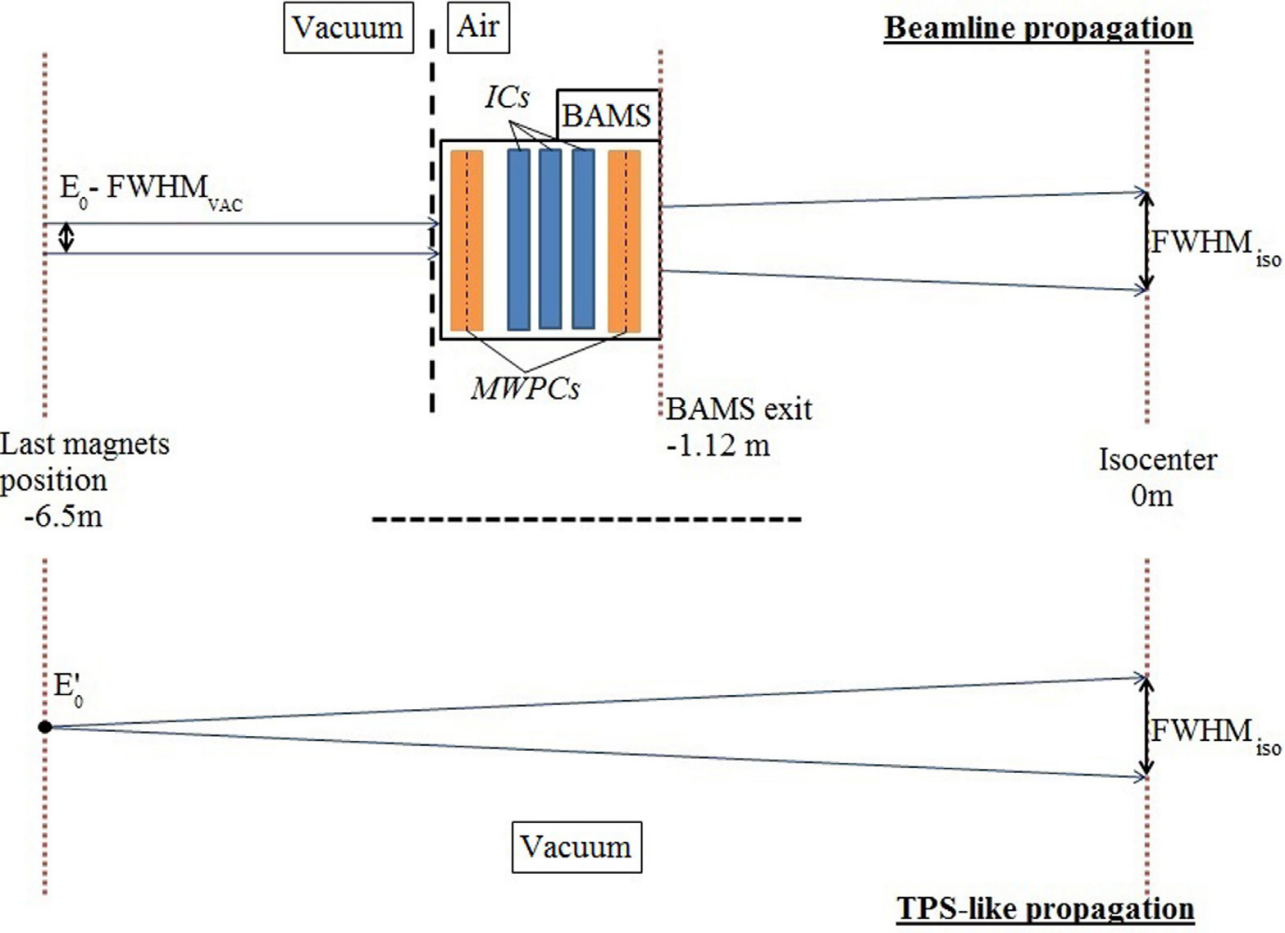
**Schematic of different MC approaches for simulation transport: on the upper panel, the detailed beamline allowing precise description of the particle interactions, from a beam with an initial energy *E*_0_ and an initial focus FWHM_vac_; on the bottom panel the simplified TPS-like approach propagating the particle in vacuum to the isocenter, with an initial energy *E*′_0_ taking into account the energy reduction of *E*_0_ due to the BAMS water equivalent thickness and adapted geometrically to the same FWHM_iso_ used by the TPS at isocenter**.

Different approaches have been used concerning the modeling of the beamline for MC simulation at HIT. The detailed geometrical model (5) allows simulating more precisely transport and interactions occurring in the beamline, particularly inside the BAMS, for accurate prediction of lateral beam scattering (Figure 1). The different foci are representative of the spread of an initially small (few millimeters) beam in vacuum into the beamline and air. In the simplified MC framework, the beamline is approximated by an energy reduction before the propagation of the particles in vacuum, according to the water equivalent thickness of the beamline and air distance to the isocenter. The focus is then adapted geometrically to its nominal one at the isocenter (8), similar to the TPS approach. With this simplified approach, forward recalculation of planned treatments could well reproduce corresponding dosimetric measurements in most of the cases, with differences below 3% (8). However, the approximations made in such MC framework [the so-called TPS-like approach (8)] could have limitation for extreme cases of small fields, due to an underestimation of large angle lateral scattering in the elements before the target. Furthermore, with the explicit modeling of the beamline geometry, information on the primary and secondary particles exiting the BAMS could be tracked, as well as their impacts. Hence, in order to give the possibility to external users to perform precise simulations using the detailed geometrical modeling of the beamline, without disclosing its confidential components, we developed an original PS approach (see Section “Phase Space Narrow-Beam Approach”).

The FLUKA version used for this study is the 2011.2c. In order to reproduce HIT reference Bragg curves, several optimizations have been made on the initial beam momentum spread for every energy as well as the ionization potential in water, similar to previous studies using older FLUKA versions (4, 20). The “HADROTHErapy” settings with the “EVAPORation” physics model were used for both PS generation and dosimetric verification. For time-efficient generation of PS files as well as data space saving, photons and electrons were not transported, thus depositing their energy at the production point.

#### Phase Space Requirements

The PS files characterize the beam on a plane perpendicular to its propagation at a defined position along the beam path, by describing the properties of every crossing particle (charge, mass, energy, coordinates and direction cosines, generation).

Several goals were defined prior to the generation of the PS. For every initial beam energy of protons and carbon ions, a unique PS should be generated and adapted to all the possible foci available at HIT. The FWHM in air at isocenter obtained with the PS should not be different from the reference foci values of the TPS basic data or LIBC (i.e., FWHM in air at isocenter) within the tolerances defined internally at HIT to account for possible daily variations of the beam shape [(−15, 25%) of the reference]. The same tolerances are defined for comparing the simulations using the new PS approach to measurements of FWHM in water at different depths. For the comparisons between the full beamline geometry (BL approach) and the PS approach, we decided that the differences in FHWM at isocenter in air should be inferior to 3%. Additional requirements include a consistent propagation of the primary and secondary particles, meaning that particles generated from the same primary history have to be transported together. Also, the PS approach should lend itself to beam propagation according to the raster scanning pattern of the treatment plan. A reasonable compromise between the size of the PS files and the number of simulated particles has to be found, in order to have enough available statistics per energy and also saving all the needed information.

#### Phase Space Narrow-Beam Approach

In order to respect the requirements on the adaptability of a unique PS to different foci, we develop an original narrow-beam approach for PS files generation. It can be explained by analogy with a homogeneous analytical system, whose response *R*_δ_ to a Dirac signal δ is its impulse response *S*. In addition, the response *R_*G*_* of this system to a Gaussian signal *G* will be the convolution between the signal *G* and the impulse response *S*.

$$R_{\delta }=S*\delta =S$$

$$R_{G}=S*G$$

In this way, when using an infinitely narrow (“zero-width”) beam propagated in the beamline (by analogy δ), the PS scored at the end of the BAMS of the beamline (by analogy the system), specifically the information on the particles position, represents the impulse response *S* of this system.

Therefore, an adaptation to every focus is possible by convoluting the PS with the information on the particle position, using a Gaussian distribution *G* to represent the beam in vacuum before entering the beamline. It is known that the result of the convolution between two Gaussian functions is still a Gaussian, with a width (standard deviation, SD) σ(G1*G2) corresponds to the quadratic addition of the widths of the two Gaussians G1 and G2, σ(G1) and σ(G2). Assuming that the fluence distribution of this PS is Gaussian-like, this approach is consistent with the quadratic addition as in Parodi et al. (5), with σ the beam focus at isocenter, σ_0_ the beam broadening at isocenter due to a “zero-width” beam and σ_ini_ the estimated initial beam in vacuum:

$$\sigma {\left(G1*G2\right)}^{2}=\sigma {\left(G1\right)}^{2}+\sigma {\left(G2\right)}^{2}$$

$$\sigma^{2}=\sigma_{0}^{2}+\sigma_{ini}^{2}$$

For every focus, a different value of the beam initial size in vacuum is needed and has to be estimated. The theoretical Gaussian FWHM of the beam in vacuum (before the beamline) is investigated as a function of the energy using this narrow-beam approach. By scoring the position of the primary particles at the isocenter, for several energies in the therapeutic range, and evaluating the FWHM of their distributions at the center of the beam spot along the horizontal axis, the FWHM of the vacuum Gaussian beam *FWHM_*Vacuum*_*(focus)** can be retrieved using the following equation for every focus:

$$\begin{array}{l} FWHM_{Vacuum}{\left(focus\right)}^{2}=FWHM_{Isocenter}{\left(focus\right)}^{2}-FWHM_{Isocenter}(\delta {)}^{2} \end{array}$$

where *FWHM_*Isocenter*_*(focus)** is the FWHM size at isocenter in air for one focus extracted from the HIT LIBC database, *FWHM_Isocenter_(δ)* is the FWHM size in air at isocenter after the propagation of an infinitely narrow beam. The energies investigated are {48.12, 54.19, 80.90, 106.82, 132.30, 157.43, 182.66, 221.05} MeV/u for protons and {88.83, 100.07, 150.42, 200.28, 250.08, 299.94, 350.84, 430.10} MeV/u for carbon ions.

The calculated values of *FWHM_*Vacuum*_*(focus)** are compared to the expected ones from previous work (4) and are used for the final validation of the PS approach as well as for the rest of the study. A total of 10 million primary histories are simulated for each run.

With the beam records of the irradiation, where the information about the size of the focus at the isocenter are recorded, a new estimated focus size in vacuum could be calculated by replacing the nominal *FWHM_*Isocenter*_*(focus)** with the one extrapolated from the upstream measurement of the BAMS.

#### Phase Space Scoring

Phase space files are generated for protons and carbon ions, for every energy of the HIT accelerator library with the optimized beam momentum spread in the simulation, transporting 10 million primary particles in total, which results in files with a total size of about 500 Mb each. The lateral size of the beam is set to a zero-width distribution (see Section “Phase Space Narrow Beam Approach”). The scoring is done on a 4 m^2^ plane perpendicular to the beam direction at the end of the BAMS, just after the last element of the beamline, i.e., the second multiwire proportional chamber, at about 112 cm before the isocenter.

Two files are created. The first file corresponds to the scoring of the primary beam with the information about the energy, the position in the plane (*X*,*Y* position), and the direction cosines (*X* and *Y* cosines). The second file contains the information about the secondary particles (except photons and electrons) in terms of energy, position, direction cosines, charge, and mass information of every particle. Last information to be saved in both files is the generation number of the primary, which allows linking primary to secondary particles during the propagation process.

To ensure that the PS is representative of the different interactions occurring in the beamline, the starting positions of the narrow beam are randomly selected in a 5 mm*5 mm square around the central axis. Information on these starting positions is kept during the beam propagation in the beamline to the scoring position, and then subtracted to the scored position of every particle in the PS files.

#### Phase Space Propagation for Scanned Beam Delivery

**Figure 2:**
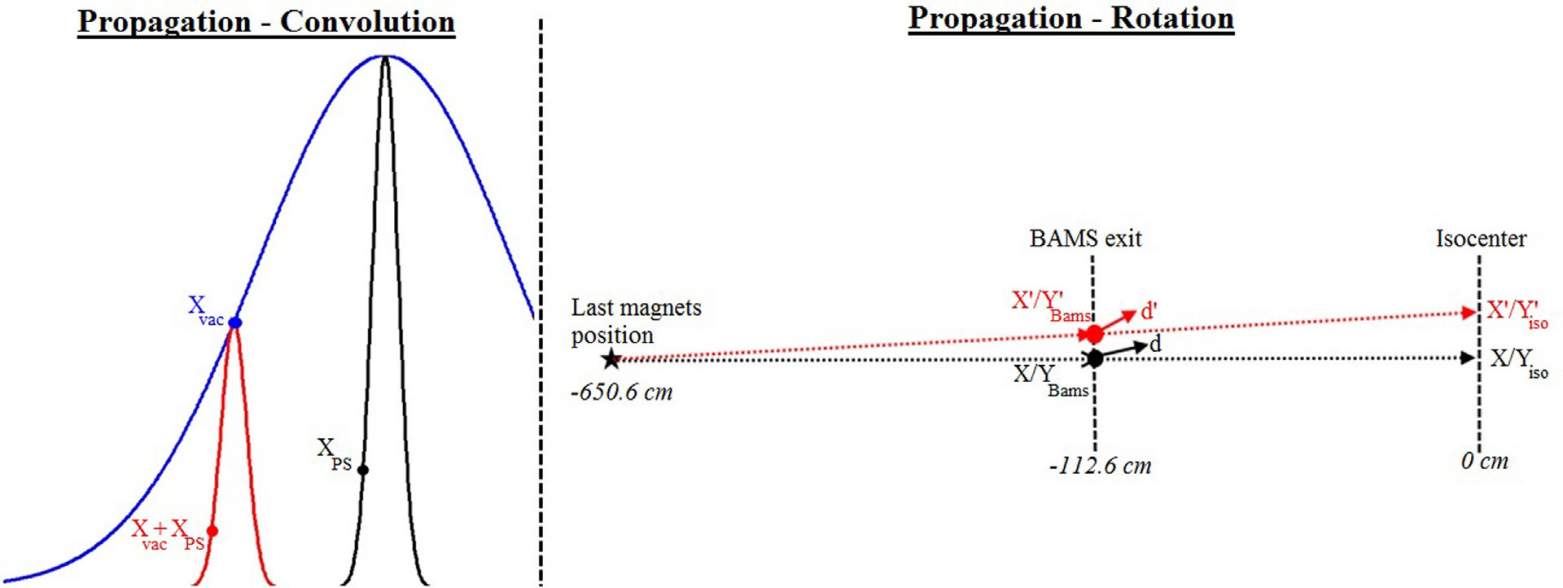
**Propagation process: on the left panel, convolution results (red) between the PS from the narrow beam (black) and the Gaussian in vacuum (blue): new position (*X*_vac_ + *X*_PS_) obtained from the original position on the phase space (*X*_PS_) and the selected position on the Gaussian in vacuum (*X*_vac_); on the right panel, rotation of the beam to the expected position: transformation from the *X*/*Y* position at the BAMS with a direction cosine d to the new *X*’_Bams_/*Y*’_Bams_ position at the BAMS with the direction cosines d’ in order to reach the *X*’_iso_/*Y*’_iso_ position at isocenter**.

While performing a treatment plan simulation using the PS, the so-called PS approach, the propagation process is divided in five steps:

- Reading the plan used for irradiation and segmenting the requested number of primary histories for the simulation run among the different energy slices according to the weight of the number of particles per slice compared to the total number of particles of the plan. This is the only step of the process that is not random in order to read the PS files only one by one, not to overload the computer memory.
- Reading the PS file of the current energy slice and linking primary and secondary particles.
- Selecting randomly a generation number among the 10 million possible, to be handled as the next primary history. Then loading all the particles related to this primary history into the stack of particles to be propagated. Every possibility can be handled (a primary with no secondaries, only secondaries, primary and secondaries, no particles scored in the PS).
- The convolution process at the single particle level is performed by adding the position information (*X*_PS_, *Y*_PS_) of each particle of the stack (loaded from the PS) to, respectively, two distinct random positions (*X*_Vac_, *Y*_Vac_) selected from a Gaussian distribution with a FWHM size corresponding to the expected Gaussian size of the beam in vacuum for the selected focus (see Figure 2 and Section “Phase Space Narrow Beam Approach”). This initial Gaussian distribution in vacuum is assumed to have the same FWHM in *X* and *Y*. The final position of the particle to be transported is then *X*_PS_ + *X*_Vac_, *Y*_PS_ + *Y*_Vac_.
- Selecting randomly one of the planned positions at isocenter (*X*’_iso_, *Y*’_iso_) in the current energy slice, with probability weighted by the number of particles to be delivered to this spot compared to the total one of the energy slice. In order to reach this position, the PSs have to be rotated from the original position (*X*_iso_, *Y*_iso_, corresponding to the position of the central beam axis at isocenter) to the selected coordinate (*X*’_iso_, *Y*’_iso_). This means finding a new position on the PS plane (*X*’_Bams_, *Y*’_Bams_) with respect to the original one along the central axis (*X*_Bams_, *Y*_Bams_) and adapting the new direction cosines (*d*’) from the original one of the selected particles (*d*) in order to target the selected isocenter position after propagation (Figure 2).

This method holds the advantage that even with only 10 million particles in the PS, the convolution with random positions of the beam Gaussian shape in vacuum increases the number of combinations of position and energy, thus, decreasing the probability to have the same event repeated twice.

### Validation and Comparisons

#### Validations of the PS Approach

Validations of the PS approach are performed against the BL approach, i.e., propagation with the beamline geometry, for different cases. The first pencil-beam validation step is focused on the differences between the two approaches in terms of fluence distributions and particle spectra for a central beam delivery without scanning. For the additional validation steps with scanned beam delivery featuring line scans and spread out Bragg-peak (SOBP) distributions, the comparisons are made on the dose results.

##### Pencil-Beam Validation

For both protons and carbon ions, two energies have been investigated, namely the lowest (respectively, 48.12 and 88.83 MeV/u) and the highest (respectively, 221.06 and 430.10 MeV/u), for the smallest and largest foci (i.e., focus indexes 1 and 4) used in clinical routine. Different PS files were generated at different positions along the beam path in order to investigate the beam propagation in air for a fixed central pencil beam: PS_BAMS_ is recorded on a 4 m^2^ plane at the BAMS exit at the same position as the one generated with the narrow-beam approach, while PS_iso_ is recorded on a 4 m^2^ plane at the isocenter. Three scenarios are compared for the different energies:

- Simulations with BL approach with different focus sizes: PS_BAMS,BL_(focus) and PS_iso,BL_(focus), starting from a beam in vacuum with the estimated beam size in vacuum before the beamline.
- Simulations with BL approach for the narrow-beam propagation: PS_BAMS,BL_(δ) and PS_iso,BL_(δ), starting from an infinitely narrow beam in vacuum before the beamline, as in the PS generation process.
- Simulations with the PS approach for different focus sizes: PS_iso,PS_(focus), starting from the PS in air at the BAMS exit position (where the PS have been generated).

The fluence and energy distributions are investigated for both primary and secondary particles.

On the planes perpendicular to the beam propagation, at the BAMS exit position and the isocenter, the FWHM of the fluence distributions at the center of the pencil-beam spot along the horizontal axis are reported, as well as the FWHM of the vertically integrated profiles.

For the vertically integrated profiles at the isocenter, the absolute global differences are also analyzed. It corresponds to

$$Difference_{global}\left(x\right)=100\times \frac{\left|Fluence_{PS}\left(x\right)-Fluence_{BL}\left(x\right)\right|}{\max\left(Fluence_{BL}\right)}$$

with *Fluence(x)* the fluence on the profile at the position *x* (for both approaches), and *max(Fluence_BL_)* the maximum fluence along the profile. The mean of these differences and its SD σ, as well as the maximal deviation, are reported. These values are calculated in a region of the profiles where the fluence is superior to 0.1% of the maximal fluence. The bin size of the profile is 0.2 mm.

For the energy spectrum, the same analysis is performed on the different PS files acquired at the isocenter, however, the *x* variable corresponds to an energy bin in the energy distribution. The bin size is 0.04 MeV/u. The energy spectrum of the secondaries is qualitatively analyzed as their proportion compared to the primaries is low (maximum probability for an energy bin around 0.05% per primaries), hence, only the trend and similarities of the spectrum are compared.

For the BL and the PS approaches, scenario 10 and 5 million primary histories are simulated, respectively. For quantitative purposes, only 5 million primary histories are used for the analysis, for both approaches, in order to have a fair comparison.

##### Line Scan Validation

For both protons and carbon ions, we designed plans corresponding to a vertical line scan, extending from −5 cm to +5 cm with a 1 mm step and centered horizontally (i.e., at 0 cm). Three initial beam energies within the therapeutic range are investigated, a low energy (80.90 and 150.42 MeV/u for protons and carbon ions, respectively), a middle energy (157.43 and 299.94 MeV/u, respectively), and the highest energy (221.06 and 430. MeV/u), in combination with each of the four foci used in clinical routine; thus, resulting in a total of 24 line scans. The geometry of the simulated target represents the water phantom used for plan verification measurements, positioned at the treatment isocenter, with a 5 mm PMMA entrance window. The bin size of the dose scoring grid is set to 0.5 mm × 0.5 mm × 0.5 mm. To ensure enough statistics, 100 million primary histories were simulated for both approaches in 100 statistically independent runs. Both laterally integrated depth dose profiles, scored along the beam penetration in water, and lateral dose profiles, sampled at the entrance of the target, are compared between the BL and PS approach. For every dose profile, we investigate both the absolute local dose relative difference:

$$Difference_{local}\left(x\right)=100\times \frac{\left|Dose_{PS}\left(x\right)-Dose_{BL}\left(x\right)\right|}{Dose_{BL}\left(x\right)}$$

and the absolute global dose relative difference:

$$Difference_{global}\left(x\right)=100\times \frac{\left|Dose_{PS}\left(x\right)-Dose_{BL}\left(x\right)\right|}{\text{max}(Dose_{BL})}$$

with *Dose(x)* being the dose of the profile at the position *x* (for both approaches), and *max(Dose_BL_)* being the maximum dose along the profile.

##### SOBP Validation

Spread out Bragg-peak plans have been simulated with both the PS and the BL approaches for protons and carbon ions, in the latter case using the ripple filter geometry (21), used to broaden the narrow Bragg peaks of carbon ions, as done in clinical practice. SOBP plans are designed to deliver 1 Gy to a 5 cm × 5 cm × 3 cm target, centered at 10 cm depth in water. The same MC geometry with the water phantom is used, as described in Section “Line Scan Validation.” The dose scoring grid is set to a bin size of 1 mm × 1 mm × 1 mm. 100 million primary histories are used to simulate these plans. In this more clinical-like scenario, only the absolute global differences of the doses between the BL and PS approaches are investigated along the central depth dose profile and for the lateral dose profiles sampled at the entrance of the target and in the middle of the SOBP.

#### Comparisons of the PS Approach with Measurements

The line scan plans, presented in Section “Line Scan Validation,” have also been irradiated at the experimental room of HIT. The measurements were performed in a water phantom coupled with 24 motorized Pinpoint ionization chambers (PTW, 0.03 cm^3^). The chambers are positioned in a block composed of six horizontal lines with four chambers (separated of 12 mm one to each other within the same line) at six depths along the beam path, separated by 10 mm. In the vertical direction, the lines are grouped by two, and these three groups of two lines are separated from each other by 7 mm. Within a same group of two lines, these two lines are shifted by 6 mm horizontally to avoid interferences from one line to the other one. This allows acquiring four positions at the same depth, i.e., for the same horizontal profile, for six depths in each measurement. Then for the same block position in depth, six measurements were performed with a 2 mm horizontal shift perpendicular to the beam direction to complete the lateral profiles. The lateral extension of each profile is 46 mm. The block was put at three positions along the beam path in order to sample lateral profiles at the entrance of the phantom, in the plateau of the Bragg peak and near the Bragg peak. The measurements were acquired for every combination of particle type/energy/focus presented previously.

For this comparison, the MC simulations using the PS approach are the same as the one presented previously. However, in order to have a fair comparison between the PS approach and the measurements, the sensitive volume of the ionization chamber has been taken into account in the MC dose results, by averaging the dose value of the voxel of interest with the surrounding ones to obtain a resulting integration volume close to the one of the ionization chamber.

The lateral profiles are analyzed quantitatively at three different depths, for each energy and focus, and the FWHM values of both measurements and simulation are compared. For the lowest energy, with a range of around 53 mm, the depths analyzed are 15.7, 30.7, and 45.7 mm. For the middle energy, with a range around 172 mm, the depths analyzed are 15.7, 85.7, and 151.7 mm. For the highest energy, with a range around 308 mm, the depths analyzed are 15.7, 195.7, and 267.7 mm. The mean and the SD of the absolute differences are reported for protons and carbon ions.

### Application to a Small Target Clinical Case

A challenging clinical entity has been selected for testing the PS application: an arterio-venous malformation (AVM) that is a small target inferior to 20 ml in most of the cases and below 3 ml in our study, treated at HIT with protons in one fraction of 18 Gy RBE at the isodose 80%. Magro et al. (11) found for small targets at shallow depth discrepancies between TPS and measurements in water up to ~19%.

Among the four beams of the plan, we selected the one delivering the highest dose. Dosimetric measurements for this beam were performed in the same water phantom described in the Section “Comparisons of the PS Approach with Measurements,” and compared to the dose calculations resulting from the PS approach and the simplified MC framework using the TPS-like approach. Several lateral profiles in the horizontal direction, with a 1 mm lateral step, are acquired at different depths of 19.7, 29.7, 39.7, and 49.7 mm.

Furthermore, using the information from the irradiation beam records registered by the BAMS, it was found that all foci were on average 1 mm larger than the ones of the TPS database, used in both the PS (in terms of the beam vacuum size added to the narrow-beam approach) and TPS-like simulations. Hence, new expected Gaussian sizes of the beam in vacuum were generated and an additional simulation was performed for the PS approach with these new parameters for comparison to the measurements and the previous simulations.

The geometry for the MC simulations is using the same water phantom target as for the SOBP simulations. The dose scoring grid is with a bin size of 1 mm × 1 mm × 1 mm and the number of primary histories is set to 5% of the beam total number of particles, which is five times higher than the recommended statistics according to Bauer et al. (8).

In a second step, forward dose calculations of the whole plan in the patient CT geometry have been performed for both the TPS-like approach and the PS one, using the reference LIBC foci value at isocenter. The results are compared in terms of dose profiles sampled within the target region [planning target volume (PTV)] region and PTV dose volume histograms.

## Results

### Validation of the PS Approach

#### Gaussian Shape in Vacuum

For carbon ions, the calculated FWHM values of the Gaussian lateral beam distribution in vacuum are within 0.2 mm to the ones expected: 2.5, 5, 7.5, and 9.5 mm for the foci from 1 to 4. For focus 1, the mean calculated FWHM (μ) is 2.46 mm with a SD σ of 0.15 mm, μ = 5.02 ± 0.09 mm, μ = 7.47 ± 0.07 mm, and μ = 9.49 ± 0.06 mm for foci 2, 3, and 4, respectively. As the focus increases, the σ decreases due to an easiest FWHM evaluation in regard to the bin size. Considering the bin size of 0.2 mm and the small difference to the expected value, the nominal values are kept for the whole work.

**Figure 3:**
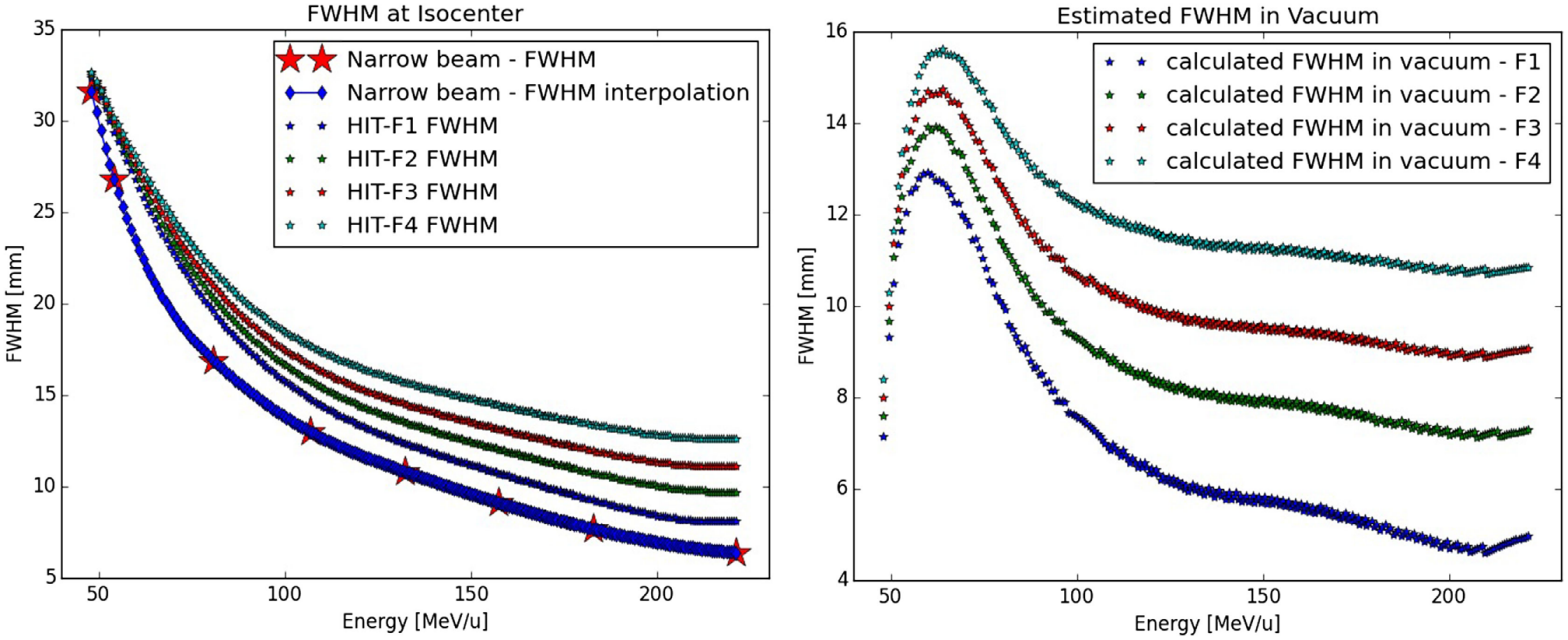
**Calculation of the protons Gaussian FWHM in vacuum: on the left panel, the different FWHM size of the proton foci at isocenter from the LIBC are displayed as a function of the energy, as well as the calculated FWHM size for the narrow-beam approach (star) and its interpolation; on the right panel, the results of the quadratic subtraction between the two previous quantities yielding the initial beam size in vacuum for the different foci as function of the energy**.

Differently, for protons the calculated FWHM values are far different from the initial values of {2.5, 6, 8, and 10} mm assumed in a previous work, which was only using a simplified beamline modeling for guiding the LIBC generation (4). It should also be reminded that for foci higher than focus 1, the FWHM foci values for the low energy region (<100 MeV/u) are not corresponding to the cited values, due to an asymptotical convergence to avoid too large beam at isocenter (4). From the simulated eight energies in the therapeutic range, an interpolation is done (Figure 3). For focus 1, we found μ = 6.46 ± 2.05 mm on the whole energy range. For energies above 100 MeV/u, for focus 2 we obtained μ = 7.69 ± 0.37 mm, for focus 3 μ = 9.40 ± 0.30 mm, and for focus 4 μ = 11.16 ± 0.26 mm. These new values are used for the whole study in order to reach with the PS simulation a good agreement to the LIBC foci values at isocenter, which are also used by the TPS.

#### Pencil-Beam Validation

##### Fluence Distributions

**Figure 4:**
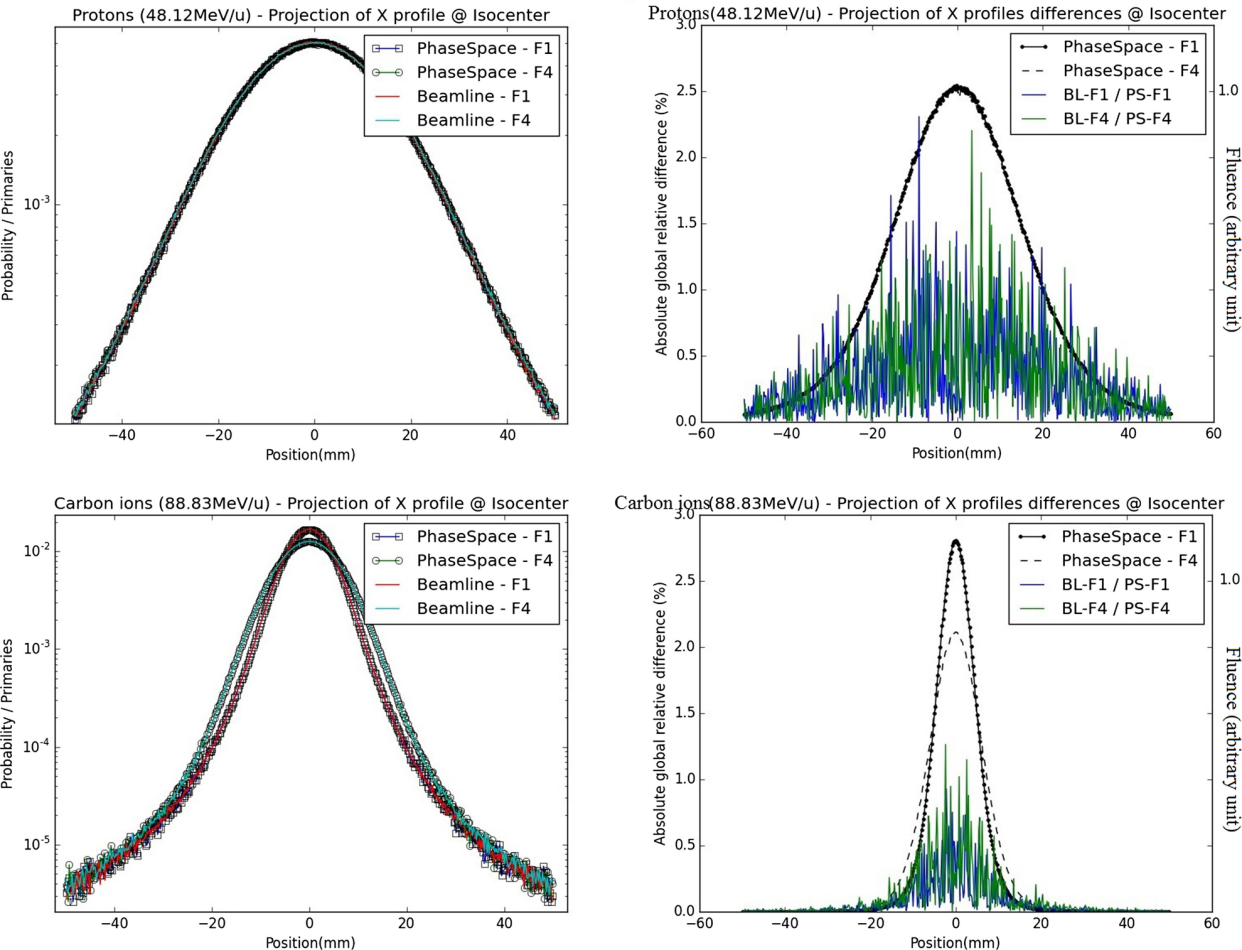
**Energy spectra difference for protons and carbon ions at the isocenter in air: on the left panels, absolute differences in primary spectra obtained with the PS and BL approaches are displayed for foci 1 and 4 (blue and red) together with the primary spectra shape (in black, similar for foci 1 and 4 and both approaches); on the right panel, secondary spectra at isocenter are displayed for the beamline approach (narrow beam and focus 1) and the PS approach; the upper panels correspond to protons, energy 48.12 MeV/u, and the bottom panels correspond to carbon ions, energy 88.83 MeV/u**.

For the two extreme foci analyzed, the lateral profiles obtained at isocenter with the PS and BL approaches are similar, regardless of the considered energy and ion species (Figure 4). The absolute global differences between the two approaches are under 2.5% for protons and under 1.3% for carbon ions (Table 1). The FWHM values of the lateral profiles, for the profiles in the center of the pencil beam spot and for the vertically integrated profiles at the BAMS exit positions and at isocenter, are reported in Table 2. For protons (for both energies and foci), the maximal difference is equal to 0.1 mm for the vertically integrated profiles and 0.2 mm for the horizontal profile along the spot center. For carbon ions (both energies and foci) the maximal difference is equal to 0.1 mm for the vertically integrated profile and 0.2 mm for the profile sampled along the spot center. For both particles type, the difference to the nominal expected values at the isocenter from the database is under 3.5% and 0.5 mm with the FWHM values in vacuum obtained from the Section “Gaussian Shape in Vacuum.”

**Table 1:** **Primary particle fluence differences at the isocenter (absolute global differences with mean μ, SD σ, and maximum value) between the PS and the BL approaches for both foci 1 and 4 for the vertically integrated lateral profile distributions, in percentages compared to the maximum fluence and in a zone of interest with an fluence >0.01% of the maximum one**.

	Protons						Carbon ions					
	48.12 MeV/u			221.06 MeV/u			88.83 MeV/u			430.10 MeV/u		
	μ (%)	σ (%)	Max (%)	μ (%)	σ (%)	Max (%)	μ (%)	σ (%)	Max (%)	μ (%)	σ (%)	Max (%)
F1	0.33	0.32	2.30	0.06	0.13	0.91	0.07	0.13	0.93	0.02	0.06	0.83
F4	0.35	0.34	2.20	0.08	0.17	1.30	0.10	0.18	1.26	0.05	0.12	0.85

**Table 2:** **FWHM, in millimeter, of the different fluence distributions for protons and carbon ions, with respect to the reference one from the LIBC: FWHM values for profiles sampled at the center of the beam spot and for vertically integrated profiles (Int. profiles), for two positions in depth in air (exit of the BAMS and isocenter) for both foci 1 and 4**.

		Position	Profiles (mm)	Int. profiles (mm)	Profiles (mm)	Int. profiles (mm)
				
			48.12 MeV/u		221.06 MeV/u	
Protons	DB-F1	Isocenter	32.4	/	8.7	/
	DB-F4	Isocenter	32.6	/	12.6	/
	BL-Narrow	BAMS	4.1	4.2	0.9	0.9
		Isocenter	32.0	34.1	6.8	6.8
	BL-F1	BAMS	8.6	8.6	5.0	5.0
		Isocenter	32.4 (0%)	34.5	8.9 (2.3%)	9.1
	BL-F4	BAMS	9.4	10.6	10.8	10.8
		Isocenter	32.5 (−0.3%)	35.0	13.0 (3.2%)	13.5
	PS-F1	Isocenter	32.3 (−0.3%)	34.6	9.0 (3.4%)	9.2
	PS-F4	Isocenter	32.6 (−0.6%)	35.0	12.8 (1.6%)	13.5
				
			**88.83 MeV/u**		**430.10 MeV/u**	
				
Carbon ions	DB-F1	Isocenter	9.8	/	3.4	/
	DB-F4	Isocenter	13.4	/	9.8	/
	BL-Narrow	BAMS	1.3	1.3	0.4	0.4
		Isocenter	9.6	9.6	2.5	2.5
	BL-F1	BAMS	2.9	2.9	2.7	2.7
		Isocenter	10.0 (2%)	10.4	3.5 (3%)	3.6
	BL-F4	BAMS	9.7	10.3	9.6	9.6
		Isocenter	13.7 (2.2%)	14.2	9.9 (1%)	9.9
	PS-F1	Isocenter	9.8 (0%)	10.4	3.5 (3%)	3.5
	PS-F4	Isocenter	13.6 (1.5%)	14.2	9.8 (0%)	9.9

*For the different foci at isocenter, the variations in percentage to the LIBC foci value are shown in bracket. DB stands for the LIBC database values, BL for the BL approach values, and PS for the PS approach*.

##### Energy Spectrum

**Figure 5:**
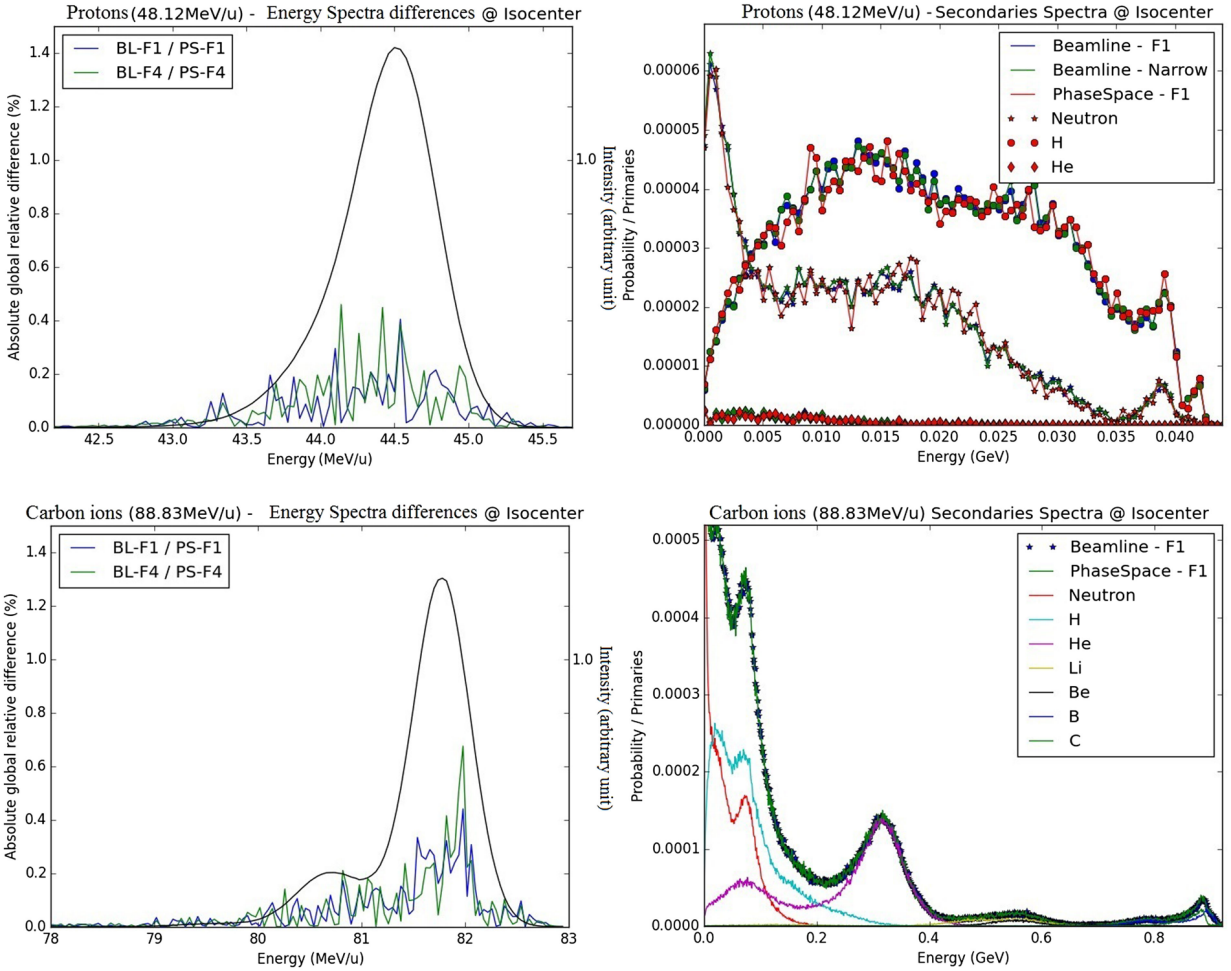
**Fluence distributions for protons and carbon ions at the isocenter in air – vertically integrated profiles: on the left panels, vertically integrated profiles (projections of X profiles) from BL and PS approaches are displayed for foci 1 and 4 in semi-logarithmic scale; on the right panels, absolute relative differences between lateral distributions from PS and BL approaches are displayed for foci 1 and 4 of the smallest energy (blue/red) together with their respective shapes (black); the upper panel corresponds to protons (48.12 MeV/u), the bottom panel corresponds to carbon ions (88.83 MeV/u)**.

From visual analysis, for the primary particles of both carbon ions and protons, the different energy spectra at the isocenter are similar for the different foci simulated with the BL (foci 1, 4, and narrow beam) or while using the PS approach for the foci 1 and 4. Quantitatively, the energy spectra at the isocenter of the BL and the PS approaches are highly similar regarding their differences (Figure 5). The absolute global differences between the BL and the PS approaches are reported in the Table 3. The maximal deviation for protons is 0.46% and for carbon ions 0.68%.

**Table 3:** **Primary particles energy spectra differences at the isocenter (absolute global differences with mean μ, SD σ, and maximum value) between the PS and the BL simulation approaches for both foci 1 and 4, in percentages compared to the maximum fluence and in a zone of interest with an fluence >0.01% of the maximum one**.

	Protons						Carbon ions					
	48.12 MeV/u			221.06 MeV/u			88.83 MeV/u			430.10 MeV/u		
	μ (%)	σ (%)	Max (%)	μ (%)	σ (%)	Max (%)	μ (%)	σ (%)	Max (%)	μ (%)	σ (%)	Max (%)
F1	0.05	0.07	0.41	0.04	0.06	0.31	0.06	0.08	0.44	0.07	0.08	0.35
F4	0.06	0.10	0.46	0.05	0.07	0.30	0.06	0.10	0.68	0.08	0.10	0.42

For the less abundant secondary particles, the different approaches show profiles with the same trend for both protons and carbon ions (Figure 5).

#### Line Scan Validation

**Figure 6:**
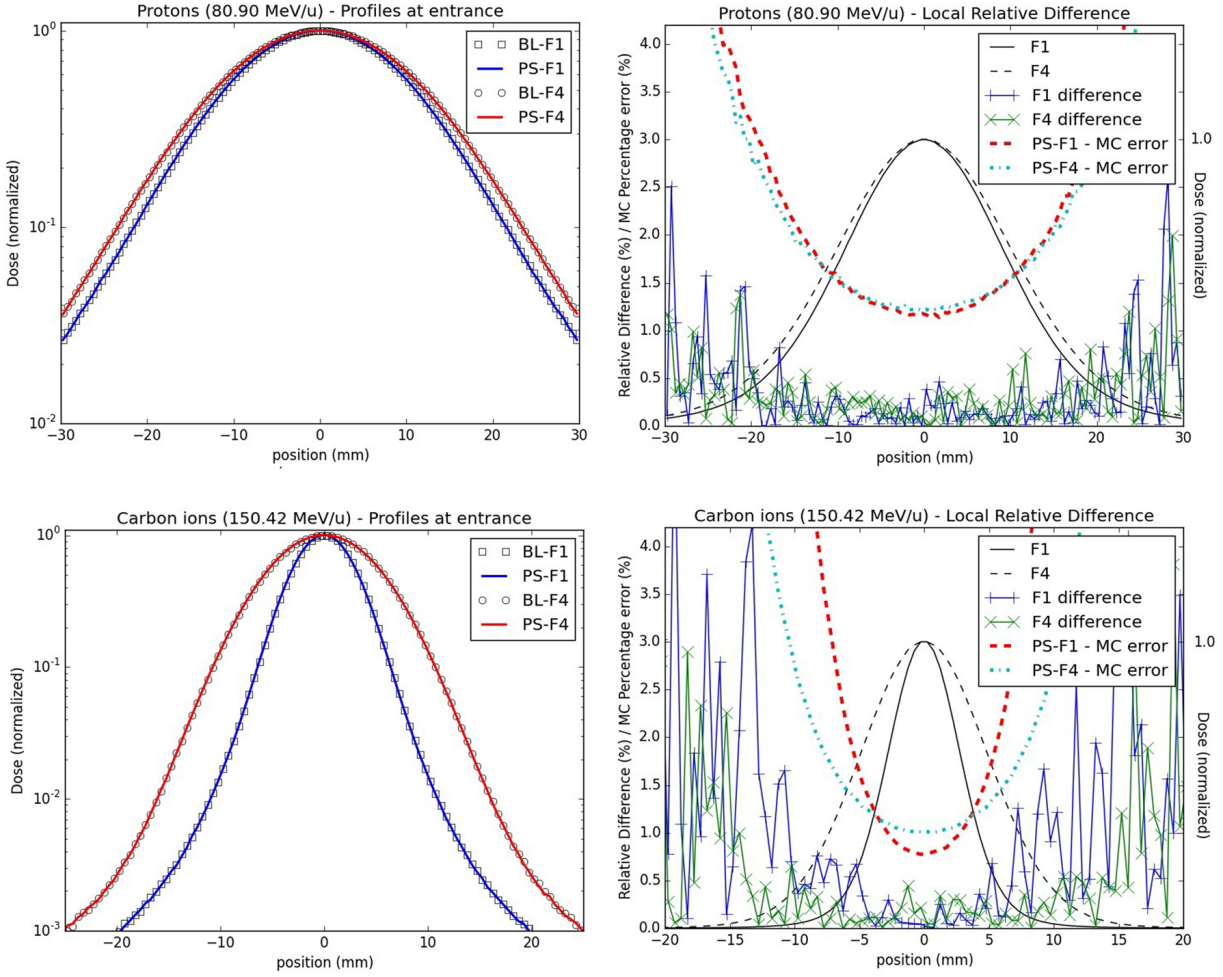
**Lateral dose profiles for line scan validations: lateral dose profiles for the lowest energy studied for both protons (upper panels, 80.90 MeV/u) and carbon ions (bottom panels, 150.42 MeV/u) at the isocenter in water; the left panels display the profiles from foci 1 and 4 for the PS (full line) and the BL approaches (stripes) in a semi-logarithmic scale; on the right panels, the profiles (black lines) together with their local relative difference (cross) and MC percentage errors (dashed lines) for foci 1 and 4 are shown**.

The line scan validation step exhibits similar results for the simulations performed with the BL and the PS approaches, both in terms of depth as well as lateral dose profiles (Figure 6).

For both types of particles and all explored combinations of energy and focus values, the absolute global dose relative difference between the PS and the BL approaches is below 0.5% for the laterally integrated depth dose profiles, and the local dose relative difference is below 0.8%. For the lateral profiles, the maximal absolute global dose relative differences are less than 0.5%, while the absolute local dose relative differences reach higher values in low dose regions, but still well below the MC percentage errors (Figure 6), as calculated over the 100 statistically independent runs.

#### SOBP Validation

**Figure 7:**
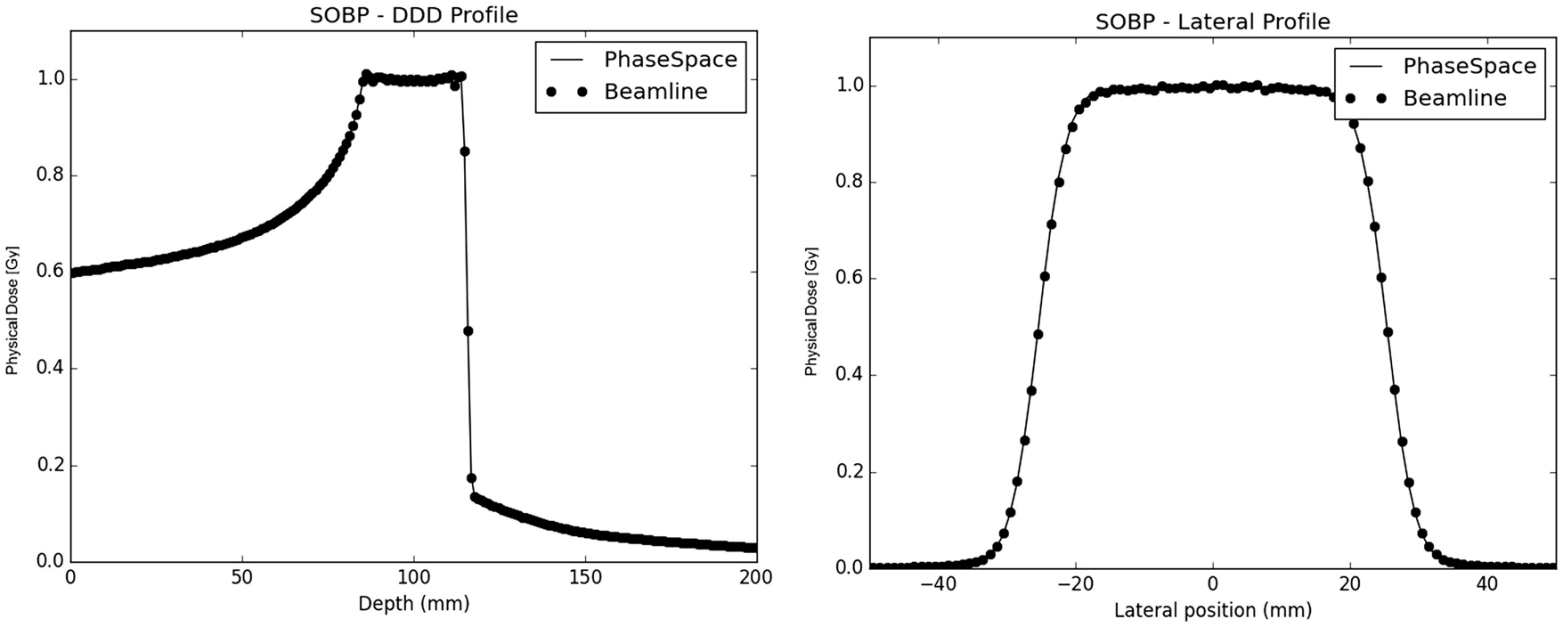
**SOBP Profiles in water: on the left panel, the depth dose profiles of both the PS (full line) and the beamline (dot line) approaches are plotted together; on the right panel the lateral dose profiles at the center of the SOBP for both the PS (full line) and the BL (dot line) approaches are shown**.

In terms of extended SOBP fields, both the simulated approaches yield depth and lateral dose profiles in excellent agreement with each other (Figure 7), with absolute global dose relative differences below under 0.5% regardless of the considered ion species.

### Comparison of PS-Based Simulations with Dosimetric Measurements

**Figure 8:**
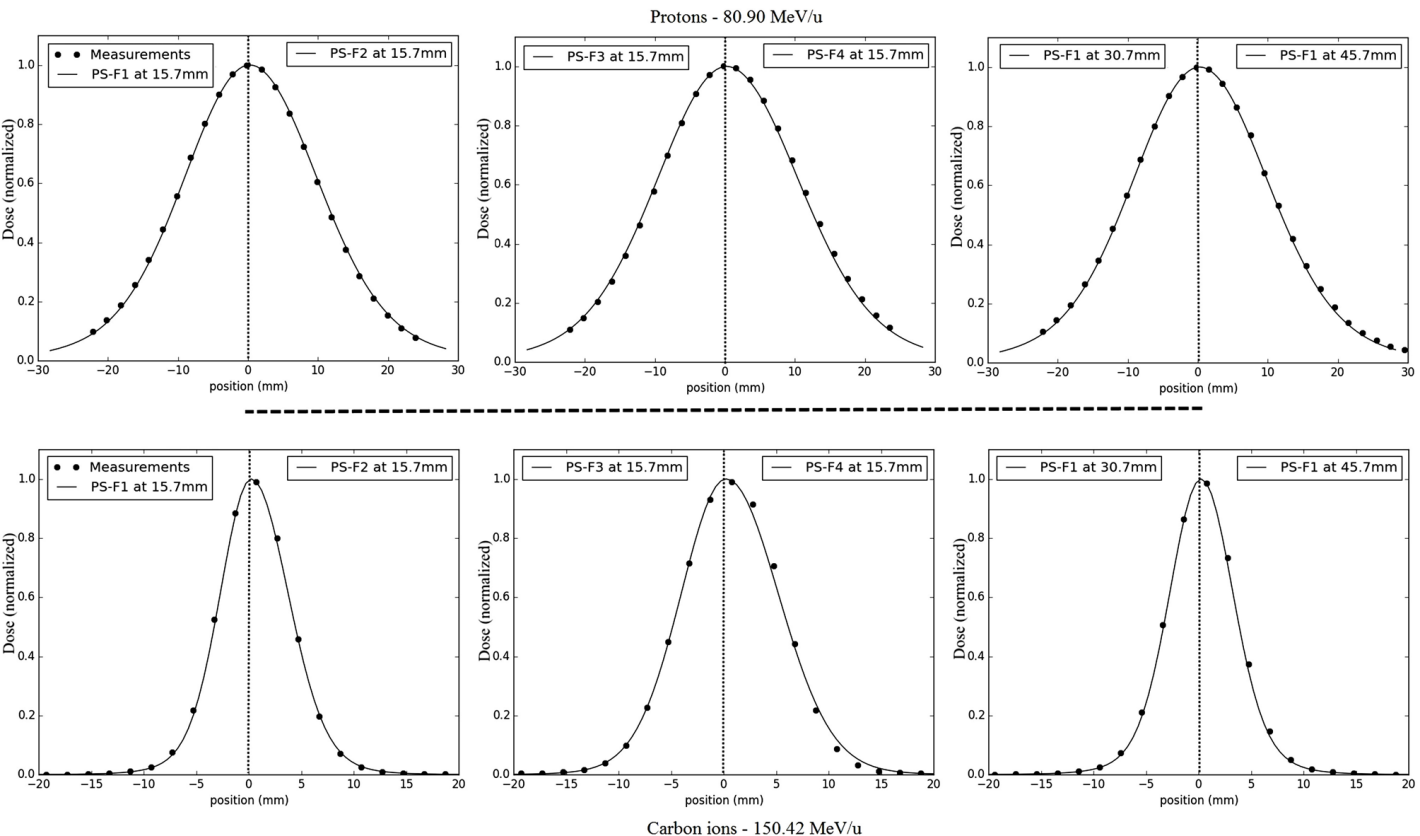
**Dose calculations and measurements for lateral dose profile comparisons from line scans: half-profiles of the different foci are plotted for the same low energy, from left to right for focus 1 to focus 4 at 15.7 mm in water, and for two other depths for focus 1 (30.7 and 40.7 mm); the upper panels correspond to protons comparisons (energy 80.90 MeV/u), the bottom panels to carbon ions comparisons (energy 150.42 MeV/u); results are normalized to the maximum**.

The different water phantom dosimetric measurements show good agreements with the PS approach simulations (Figure 8). In terms of lateral profiles sampled at three different depths in water, the differences (in mm and percentage) of the fitted FWHM values are displayed in Table 4 for both particles types, and all investigated combinations of energies/foci. The maximal relative FWHM differences found for protons are about 6.5 and 5.8% corresponding, respectively, to FWHM differences of 0.8 and 1.1 mm. The mean absolute difference of the absolute value is 0.5 mm with a SD of 0.3 mm. The maximal absolute difference found for carbon ions is of −0.9 mm (relative difference of −7.5%), the mean absolute difference is 0.2 mm with a SD of 0.2 mm.

**Table 4:** **FWHM differences, in millimeter and percentages, between the PS simulation approach and dosimetric measurements, at different depths in water, for all investigated combinations of particles/energies/foci**.

		Protons				Carbon ions			
		
	Depth (mm)	F1	F2	F3	F4	F1	F2	F3	F4
Low energy	15.7	−0.2 mm, −1.0%	0.5 mm, 2.2%	0.4 mm, 1.8%	−0.9 mm, −3.7%	−0.4 mm, −5.7%	−0.2 mm, −1.8%	−0.2 mm, −1.5%	−0.3 mm, −2.1%
	30.7	−0.3 mm, −1.3%	0.4 mm, −1.5%	−0.3 mm, −1.1%	−0.8 mm, −3.1%	−0.3 mm, −3.5%	0.0 mm, 0.0%	0.0 mm, 0.0%	−0.1 mm, −0.1%
	45.7	−0.5 mm, −2.2%	0.0 mm, 0.0%	0.2 mm, 0.8%	−0.7 mm, −2.8%	−0.2 mm, −3.2%	0.1 mm, 0.9%	−0.1 mm, −0.5%	−0.2 mm, −1.4%
Middle energy	15.7	−0.1 mm, −0.7%	0.2 mm, 1.2%	0.4 mm, 3.1%	−0.5 mm, −2.8%	−0.1 mm, −2.8%	0.1 mm, 2.0%	−0.2 mm, −1.7%	−**0.9 mm**, −**7.5%**
	85.7	0.3 mm, 2.1%	0.4 mm, 2.7%	0.6 mm, 3.5%	0.2 mm, 1.0%	0.3 mm, 6.6%	0.4 mm, 5.44%	0.0 mm, 0.0%	−0.7 mm, −5.7%
	151.7	0.3 mm, 2.0%	0.8 mm, 5.0%	0.8 mm, 4.4%	0.5 mm, 2.6%	0.2 mm, 2.7%	0.4 mm, 6.1%	0.3 mm, 3.6%	−0.1 mm, −1.3%
High energy	15.7	0.3 mm, 2.9%	0.4 mm, 4.1%	**0.8 mm, 6.5%**	0.2 mm, 1.3%	0.2 mm, 3.7%	0.1 mm, 2.0%	0.0 mm, 0.0%	−0.1 mm, −1.1%
	195.7	0.3 mm, 2.4%	0.7 mm, 4.6%	0.1 mm, 0.3%	0.9 mm, 5.2%	−0.1 mm, −1.5%	0.1 mm, 1.6%	0.1 mm, 1.0%	−0.3 mm, 2.7%
	267.7	0.3 mm, 1.8%	0.7 mm, 3.8%	**1.1 mm, 5.8%**	0.9 mm, 4.5%	−0.1 mm, −1.1%	0.3 mm, 4.0%	0.4 mm, 4.4%	0.1 mm, 1.0%

*Measurements are the reference FWHM. “Low energy” corresponds, respectively, for protons and carbon ions, to 80.90 and 150.42MeV/u, “middle energy” to 157.43 and 299.94 MeV/u, and “high energy” to 221.06 and 430.10 MeV/u*.

*The numbers in bold correspond to the most extreme variation observed for protons and carbon ions*.

### Application to a Small Target Clinical Case

**Figure 9:**
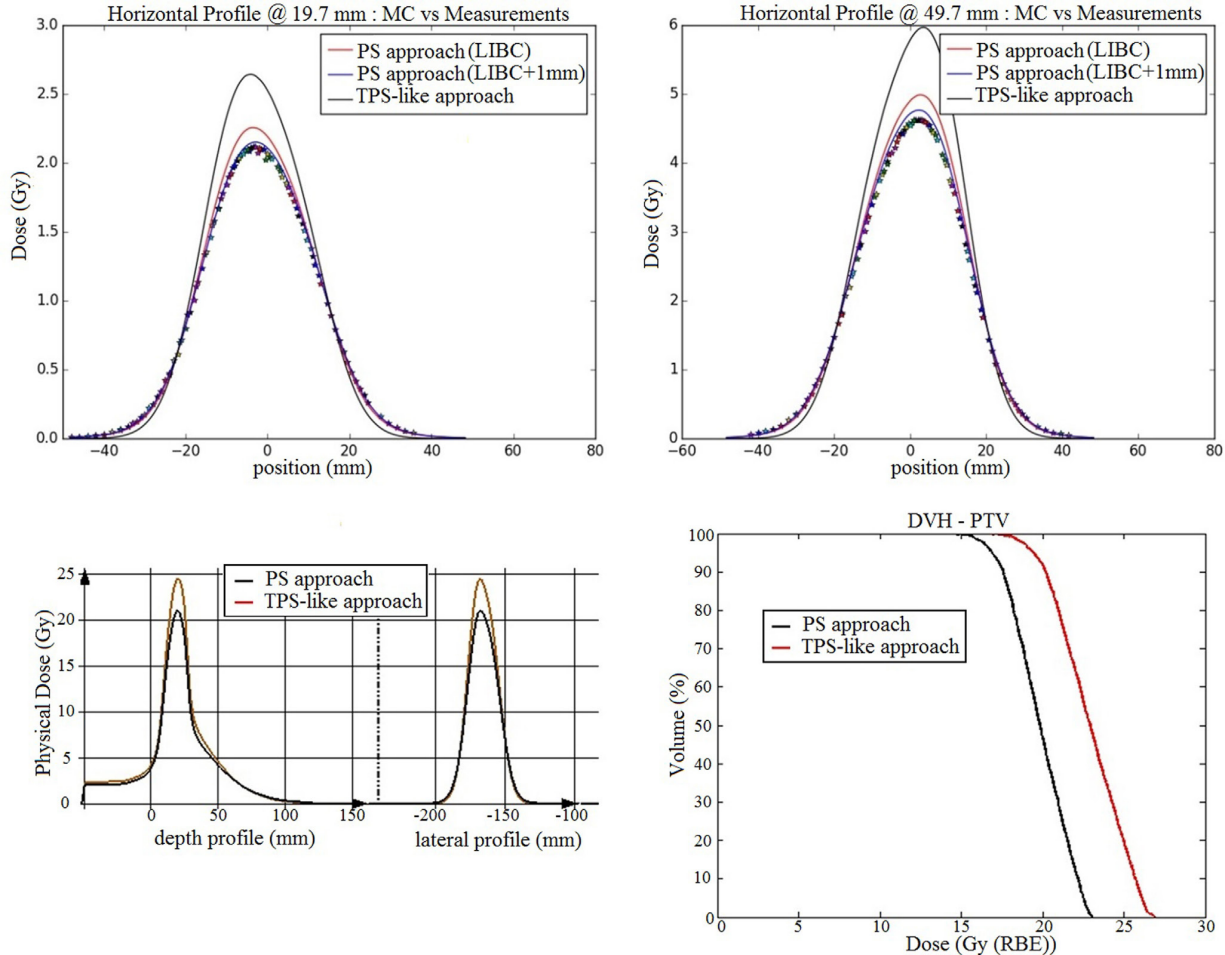
**PS approach against TPS-like approach for a small target clinical case: on the upper panels, dose verification measurements (stars) are displayed against simulations with the TPS-like approach using the database foci values (black), the normal PS approach (red) with the foci in vacuum estimated from the database foci values, and the modified PS approach (blue) using the foci values in vacuum calculated from the beam records, at two different depths in water (19.7 and 49.7 mm); on the bottom left panel, the dose profile at the PTV level in the CT patient geometry is plotted in red for the TPS-like approach and black for the PS approach, using the reference LIBC foci, for one beam; the bottom right panel displays dose volume histograms of the dose calculated with the two studied approaches**.

The comparison of the measurements acquired at different depths in water exhibits absolute global differences below 6% with the conventional PS approach (i.e., utilizing the beam width in vacuum discussed in the Section “Gaussian Shape in Vacuum”), while under 2% with the optimized PS approach which takes into account the actual deviation of +1 mm for the delivered foci with respect to the nominal TPS (LIBC) values. In such extreme scenario, the TPS-like approach implemented in the MC framework, using the nominal TPS FWHM values, yields deviations up to 25% (Figure 9).

The results of the dose calculations, using the nominal FWHM values at isocenter, for the full plan projected on the CT patient geometry show the same tendency in terms of the lateral profiles and dose volume histogram (Figure 9). Specifically, the main findings can be summarized as follows:

- 95% of the volume receives 16.9 Gy for PS approach against 19.44 Gy for the TPS-like one.
- 5% of the volume receives 22.5 Gy for PS approach against 26.1 Gy for TPS-like one.

## Discussions

### Validation of the PS

Monte Carlo simulations using the proposed PS approach show an overall very good (typically within 0.5% for the absolute global dose difference) agreement to the approach implementing the explicit modeling of the beamline.

The initial sampling of the infinitely narrow beam randomly spread within a 5 mm × 5 mm area before the beamline allows to include into the PS the information on different interactions that can occur in the beamline, particularly in the multiwire proportional chambers where the wires are separated by a 1 mm distance. Without this sampling, the spectra of the particles would have been different between the PS and the BL approaches, since some particles of the narrow-beam approach would not interact as expected with the beamline. This would also lead to deviations in terms of fluence distribution and dose deposition due to wrong direction cosines, reducing the FWHM for the fluence distributions of the PS approach, and thus resulting in a higher dose deposition in the center of the beam spot. The energy spectra of the particles obtained with the BL approach were found very similar for both foci 1 and 4, regardless of the sampling position at the end of the BAMS or at the isocenter. This means that either with a small or a large FWHM Gaussian size in vacuum, there is no major impact on the energy spectra, thus, confirming that the used initial sampling area 25 mm^2^ is adequate. Furthermore, while investigating in more details the impact of the sampling area, new PSs were generated for a 100 mm^2^ area. The fluence distribution comparison between the original and new PS did not show any relevant differences. No major differences were found for the director cosines distribution or the energy spectra either for the PS generated with the different foci and PS generated with the different sampling area, since in all these cases the initial beam is large enough to cover the multiwire proportional chambers pattern, where the wires are separated by 1 mm in the horizontal and vertical directions.

The absolute differences for the vertically integrated fluence distributions, obtained for the pencil-beam validation, are mainly due to the bin size and the resulting lower statistics per bin, since when changing the bin size from a 0.2–0.6 mm, the maximal differences drop from 2.5–0.6% for protons and from 1.3 to 0.5% for carbon ions.

The dose differences between the PS and the BL simulation approaches for the line scans and the SOBP validation are within the statistical uncertainties.

These results show that we could fulfill the initial requirements on the adaptation of a unique PS to the different foci and the consistency of the propagation starting from the PS sampling plane, including the handling of the raster scanning process.

### Comparisons of the PS-Based Simulations to Dosimetric Measurements

The overall agreement of the PS approach simulations to the lateral profile measurements in water is good, with a maximal FWHM deviation of −7.5% or 1.1 mm for the extreme cases and a mean deviation below 0.5 mm, which is corresponding to the bin size. These results are deemed as highly acceptable, taking into account the even larger tolerance of experimental foci deviations at HIT, which is from +25% to −15%.

For larger foci, particularly for carbon ions, the measured profiles exhibit asymmetric shapes in the horizontal directions, which are not modeled in our simulation. This shape is the resulting effect of the knock-out extraction process of the beam in the synchrotron, occurring in the horizontal plane, which is of trapezoidal shape (22). However, its effects are smeared out due to the scattering in the beamline, air, and water, particularly for small foci and lower energies.

### Application to a Small Target Clinical Case

The PS approach shows good results compared to dosimetric measurements in the water phantom, with an acceptable maximal deviation of 5.8%, taking into account uncertainties in the dose gradient for such an extreme case of a small target volume. Moreover, our findings also prove the power of the PS approach to adapt easily to the “real” conditions of irradiation as monitored by the BAMS, improving significantly the results. In particular, we show that MC simulations with the PS approach can use the record of the irradiation to refine from the measured foci the estimate of the actual beam size in vacuum for each energy slice. Combined with the approach used in Tessonnier et al. (23), using the measured positions of every single raster scanning spot and its associated number of particles, it could provide a powerful tool for forward calculation closer to the “real” irradiation conditions.

**Figure 10:**
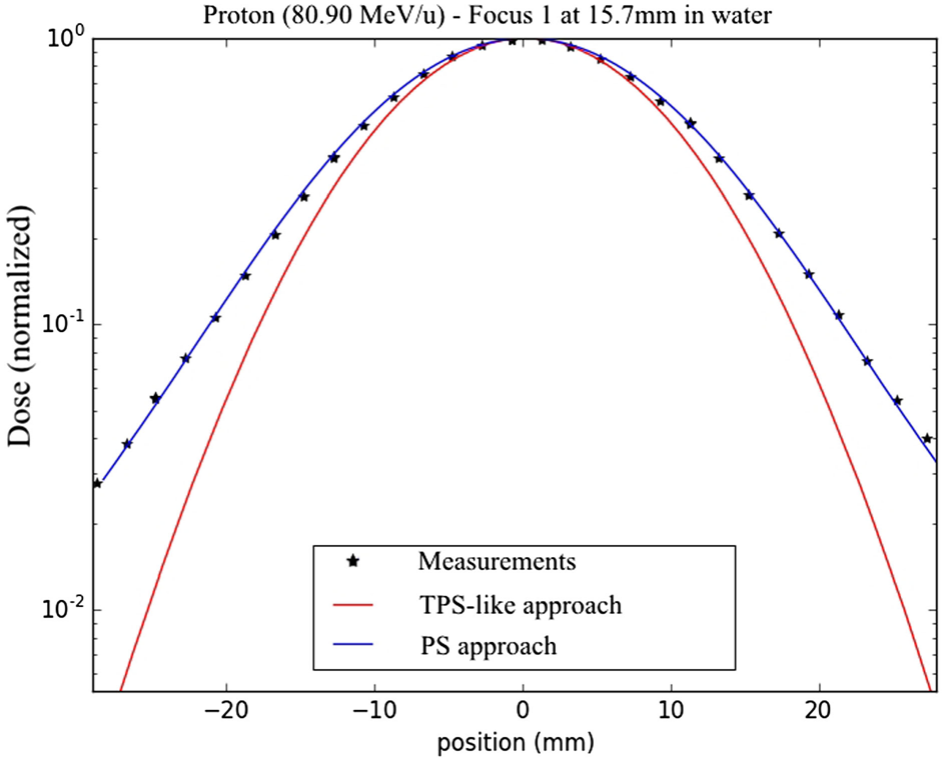
**Dose results and measurements at 15.7 mm in water, for lateral dose profiles from line scans for the lowest energy protons and focus 1 with a semi-logarithmic scale; the TPS-like approach is displayed in red, the PS approach in blue and the measurements with the star dots**.

On the other hand, the simplified TPS-like approach of the MC framework exhibits a large overestimation of the dose with a smaller size of the irradiated volume. This is because it underestimates the large angle spread of the beam due to the BAMS and the air between the end of the beamline and the target position, resulting in higher dose values in the center of every spot. This is shown in the comparison for the lowest beam energy and focus for protons used in the line scan comparisons between the new PS approach, the TPS-like one and measurements (Figure 10). These results show that beyond the accurate transport of particles in the target, the initial conditions of the beam are also fundamental. This observation is consistent with the results of Magro et al. (11) between the same TPS and MC simulations for small targets at shallow depths. Beamline approximations used for MC simulations are giving, in general, good results, as shown in Bauer et al. (8) for the MC framework, where the differences between simulations and measurements are in average below 3%, or in Grassberger et al. (12) where their model compared to a full beamline propagation show differences inferior to 1% in the middle of a SOBP. However, a precise model is fundamental for extreme cases of small targets sensitive to the exact modeling of the few individual pencil beams.

## Conclusion

A novel PS approach has been successfully introduced and validated against simulations with the full beamline geometry. It provides an accurate description of the beam to be propagated to a target (phantom/patient) as it includes the information of the interaction in the beamline in a generic way (the so-called narrow-beam approximation), allowing adaptation to different beam foci with the same data. The PS approach could bring significant improvement to the dose calculation compared to the simplified approach implemented in the current MC framework for consistency to the TPS approach, especially for the here investigated extreme situation of a small target at shallow depths.

The generated PSs can be made available for external teams upon request.

The implementation of the PS approach in the MC framework and generation of PS files for the other particles (helium and oxygen ions) available at HIT are underway.

## Author Contributions

TT conducted the work, developed the phase space approach, participated in the generation and propagation of the phase spaces with the FLUKA Monte-Carlo code as well as the different simulation, and participated in the experimental measurements. TM participated in the phase space propagation development. AM participated in every Monte-Carlo FLUKA-related tasks (phase space generations and simulations). SB participated in experimental measurements. KP supervised the whole work.

## Conflict of Interest Statement

The authors declare that the research was conducted in the absence of any commercial or financial relationships that could be construed as a potential conflict of interest.
